# Catalase, Glutathione Peroxidase, and Peroxiredoxin 2 in Erythrocyte Cytosol and Membrane in Hereditary Spherocytosis, Sickle Cell Disease, and β-Thalassemia

**DOI:** 10.3390/antiox13060629

**Published:** 2024-05-22

**Authors:** Daniela Melo, Fátima Ferreira, Maria José Teles, Graça Porto, Susana Coimbra, Susana Rocha, Alice Santos-Silva

**Affiliations:** 1 UCIBIO–Applied Molecular Biosciences Unit, Laboratory of Biochemistry, Department of Biological Sciences, Faculty of Pharmacy, University of Porto, 4051-401 Porto, Portugal; danielamelo94@hotmail.com (D.M.); assilva@ff.up.pt (A.S.-S.); 2 Associate Laboratory i4HB, Institute for Health and Bioeconomy, Faculty of Pharmacy, University of Porto, 4051-401 Porto, Portugal; 3 Hematology Service, Centro Hospitalar e Universitário de São João, 4200-319 Porto, Portugal; u007218@chsj.min-saude.pt; 4 Laboratory Hematology Service, Santo António Hospital, Centro Hospitalar do Porto, 4099-001 Porto, Portugal; mjteles.hematologia@chporto.up.pt; 5 Imuno-Hemotherapy Service, Santo António Hospital, Centro Hospitalar do Porto, 4099-001 Porto, Portugal; gporto@ibmc.up.pt; 6 Center for Predictive and Preventive Genetics (CGPP), Institute for Molecular and Cellular Biology (CGPP/IBMC), 4200-135 Porto, Portugal; 7 Abel Salazar Institute of Biomedical Sciences (ICBAS), University of Porto, 4050-313 Porto, Portugal; 8 1H-TOXRUN–One Health Toxicology Research Unit, University Institute of Health Sciences, CESPU, CRL, 4585-116 Gandra, Portugal

**Keywords:** catalase, glutathione peroxidase, peroxiredoxin 2, hereditary spherocytosis, sickle cell disease, β-thalassemia, oxidative stress

## Abstract

Catalase (CAT), glutathione peroxidase (GPx), and peroxiredoxin 2 (Prx2) can counteract the deleterious effects of oxidative stress (OS). Their binding to the red blood cell (RBC) membrane has been reported in non-immune hemolytic anemias (NIHAs). Our aim was to evaluate the relationships between CAT, GPx, and Prx2, focusing on their role at the RBC membrane, in hereditary spherocytosis (HS), sickle cell disease (SCD), β-thalassemia (β-thal), and healthy individuals. The studies were performed in plasma and in the RBC cytosol and membrane, evaluating OS biomarkers and the enzymatic activities and/or the amounts of CAT, GPx, and Prx2. The binding of the enzymes to the membrane appears to be the primary protective mechanism against oxidative membrane injuries in healthy RBCs. In HS (unsplenectomized) and β-thal, translocation from the cytosol to the membrane of CAT and Prx2, respectively, was observed, probably to counteract lipid peroxidation. RBCs from splenectomized HS patients showed the highest membrane-bound hemoglobin, CAT, and GPx amounts in the membrane. SCD patients presented the lowest amount of enzyme linkage, possibly due to structural changes induced by sickle hemoglobin. The OS-induced changes and antioxidant response were different between the studied NIHAs and may contribute to the different clinical patterns in these patients.

## 1. Introduction

Red blood cells (RBCs) are enucleated cells rich in hemoglobin (Hb), a crucial protein for oxygen (O_2_) delivery to the body’s tissues. Each day, about 3% of Hb suffers auto-oxidation, producing superoxide anions (O_2_^●−^) and methemoglobin (metHb) [1]. The accumulation of metHb and hydrogen peroxide (H_2_O_2_), resulting from O_2_^●−^ dismutation, can lead to the production of more potent reactive oxygen species (ROS) [2]. 

To counteract the damaging effects of ROS, several antioxidant agents are part of the efficient RBC antioxidant system, namely, glutathione (GSH), vitamins C and E, and the enzymes superoxide dismutase (SOD), catalase (CAT), glutathione peroxidase (GPx), and peroxiredoxin 2 (Prx2) [3]. CAT detoxifies exogenous and/or endogenous high levels of H_2_O_2_, whereas GPx and Prx2 function as scavengers of low H_2_O_2_ levels [4,5,6,7]. GPx and Prx2 can also reduce hydroperoxides and peroxynitrites [8,9]. These three peroxidases are typically characterized as cytosolic enzymes, yet many studies [5,10,11,12,13,14] have reported their linkage to the RBC membrane under oxidative stress (OS) and/or pathological conditions. 

Hereditary non-immune hemolytic anemias (NIHAs) are caused by genetic defects leading to changes in RBC membrane protein composition, in Hb synthesis or composition, and in RBC enzyme activity, and are strongly associated with OS. Hereditary spherocytosis (HS) is caused by mutations in genes encoding one of the RBC membrane proteins [15]. These alterations lead to membrane destabilization, which favors membrane vesiculation and, thereby, the formation of spherocytes [16]. Studies with RBCs from HS patients have shown several oxidative injuries, namely, increased ROS [17], lipid peroxidation (LPO) [10,17], and membrane-bound hemoglobin (MBH) levels [10,11,18]; increased binding of CAT [12], Prx2 [11], and, more recently, GPx to the RBC membrane has also been reported [10]. Splenectomy, performed in the more severe cases of HS, usually corrects anemia, hyperbilirubinemia, and the need for blood transfusions by increasing the lifespan of the RBCs [19].

Sickle cell disease (SCD) is a genetic disorder resulting from a point mutation in the β-globin gene [20]. The mutated Hb tetramers of sickle Hb (HbS) polymerize within the cell, leading to erythrocyte sickling [21], which is associated with increased cellular rigidity and reduced deformability of the RBCs, favoring premature hemolysis and the development of chronic hemolytic anemia [22,23]. Evidence of chronic OS in RBCs from SCD patients has been reported, namely, impairment of the GSH system [24], low levels of SOD and CAT [25], high LPO levels [25,26], and increased MBH [27]. 

β-thalassemia (β-thal) is caused by the absence or reduction of β-globin synthesis, leading to ineffective erythropoiesis [28]. The accumulation of unpaired α-globin chains leads to abnormal α4Hb, which precipitates in the RBCs, forming hemichromes at the membrane. Damage in the membrane will accumulate, leading to premature destruction of RBCs [29,30].

How RBCs from different NIHA patients face oxidative challenges along their lifespans is still poorly elucidated, as well as the role of OS in the several changes observed in the pathologic RBCs of the different NIHAs. 

The present study aimed to perform a comparative analysis of the antioxidant system’s response in the RBCs from patients with NIHAs–HS (unsplenectomized and splenectomized patients), SCD, and β-thal, and from healthy individuals. It also focused on the relative importance of these defense mechanisms in the erythrocyte membrane. To achieve this, we studied separately the erythrocyte cytosol and membrane, as well as overall systemic levels, evaluating the enzymatic activities and/or the amounts of CAT, GPx, and Prx2. The non-enzymatic antioxidant GSH (and its oxidized form, GSSG), ascorbic acid (AA), and other biomarkers of OS, such as, MBH, LPO, and total antioxidant status (TAS), were also assessed. To characterize the studied NIHA populations, general hematological parameters, bilirubin levels, and cryohemolysis tests were performed. 

## 2. Materials and Methods

### 2.1. Subjects

The ethic committees of the partner hospitals (Centro Hospitalar e Universitário de São João, Porto, and Santo António Hospital from Centro Hospitalar do Porto) approved the protocol used in this study, which was performed in accordance with the Declaration of Helsinki [2020.177 (138-DEFI/140-CE)]. Informed consent to participate in the study was given by all participants or by their legal representative.

A total of 93 individuals were studied, including 34 healthy individuals (the control group) and 59 subjects diagnosed with NIHAs: 22 unsplenectomized (unspl) patients with HS, 10 splenectomized (spl) patients with HS, 7 patients with SCD (7 homozygous SCD (S/S), and 20 patients with β-thal (all minor trait). All patients had been previously diagnosed and had not receive blood transfusions 4 months prior to this study. Selection and invitation of patients to participate in the study were performed by the hematologists of the team and blood collection was performed during their routine follow-up appointments. 

Groups were matched for sex (male/female: 47%/53%, 50%/50%, 40%/60%, 14%/86%, and 50%/50% for control, unspl HS, spl HS, SCD, and β-thal groups, respectively) and age (26 ± 2, 24 ± 5, 26 ± 5, 17 ± 5, and 32 ± 6 for control, unspl HS, spl HS, SCD, and β-thal groups, respectively).

### 2.2. Hematological and Biochemical Studies

The evaluation of RBC count, Hb concentration, hematocrit (HCT), mean corpuscular volume (MCV), mean corpuscular hemoglobin (MCH), mean cell hemoglobin concentration (MCHC), red cell distribution width (RDW), and reticulocyte counts were performed using an automatic blood cell counter (Sysmex XN-9000^®^, Norderstedt, Germany). The reticulocyte production index (RPI) was calculated according to Hillman [31]. Total bilirubin concentration was quantified by routine analytical procedures (Olympus AU5400 Clinical Chemistry Analyzer, Beckman Coulter, Brea, CA, USA).

### 2.3. Blood Samples 

Blood samples were collected (EDTA as anticoagulant, 5 mL) by venopuncture; one aliquot (500 µL) was used to perform the cryohemolysis test (CHT). To perform this test, RBCs isolated after centrifugation (1000× *g*, 4 °C, 5 min) were used and washed with saline solution. The percentage of hemolysis was calculated as described by Streichman et al. [32].

The remaining whole blood was centrifuged (2000× *g*, 4 °C, 10 min) to obtain plasma, which was stored at −80 °C until assayed; the pellet (blood cells) was used to isolate RBCs that were further processed to obtain two fractions, namely the erythrocyte cytosol and the erythrocyte membrane. 

The RBC cytosol and membrane fraction samples were prepared after isolation of RBCs through a double density gradient (Histopaque 1.077 and 1.119, Sigma-Aldrich, St. Louis, MO, USA) centrifugation (700× *g*, RT, 30 min), discarding leukocytes. RBCs were washed in saline solution and, afterwards, aliquots (50.0 µL) were incubated with or without 100 mM N-ethylmaleimide (NEM) (10 min, RT, gentle shaking). NEM was added to preserve the original redox forms. To finally obtain the RBC cytosol fraction, the samples were submitted to hypotonic lysis with Dodge buffer [33], containing the protease inhibitor phenylmethylsulfonyl fluoride (PMSF, 0.1 mM). The supernatant (cytosolic fraction) was collected after centrifugation (20,000× *g*, 10 min, 4 °C) and stored at −80 °C until assayed. The cytosol fractions incubated with NEM were used for Prx2 immunoblots, and the samples without NEM were used for the other assays. 

The remaining RBCs were used to prepare the RBC membrane fraction, as previously described [10]; the obtained RBC membrane samples were stored at −80 °C until assayed. 

Before performing the analytical studies on the membrane or the cytosol samples, Bradford’s method [34] was performed to determine the concentration of total protein. The values obtained in all of the following assays were normalized to the protein concentration of each sample/fraction.

### 2.4. Antioxidant Enzyme Activities

CAT activity was measured in plasma, cytosol, and membrane samples using a spectrophotometric method, according to Johansson et al. [35]. Briefly, methanol and H_2_O_2_ (35 mM) were added to the samples. After incubation (20 min, RT, under gentle shaking), the chromogen Purpald (70 mM) was added, and the formaldehyde produced (proportional to CAT activity) was measured at 540 nm. A formaldehyde standard curve was used. 

GPx activity was measured in plasma and cytosol by an indirect spectrophotometric assay, according to Weydert and Cullen [36], by which NADPH oxidation by glutathione reductase was monitored at 340 nm. The rate of decrease in the absorbance at 340 nm was directly proportional to GPx activity. This GPx assay was not sensitive enough to quantify GPx activity in membrane samples. 

### 2.5. CAT, GPx, and Prx2 Amounts 

CAT, GPx, and Prx2 amounts were evaluated in cytosol and membrane samples by enzyme-specific immunoblots. Cytosol and membrane samples were treated with an equal volume of solubilization buffer [0.125 M Tris–HCl at pH 6.8, 4% sodium dodecylsulfate (SDS), 20% glycerol, 10% 2-mercaptoethanol, and 0.1% bromophenol blue] and heat-denatured. A solubilization buffer without the reducing agent (2-mercaptoethanol, non-reducing conditions) was used to prepare the samples (previously incubated with NEM) for the Prx2 immunoblots. 

For quantification of CAT and GPx in the RBC membrane (30 μg protein/lane) and cytosol (250 μg protein/lane) samples, we performed SDS-PAGE using a 5–15% linear acrylamide gradient gel [37]. Afterwards, the proteins were transferred to a nitrocellulose sheet (0.20 µm) and incubated for 1 h in 5% low-fat milk and 0.1% Tween-20 in tris-buffered saline (TBS), pH 7.4. 

For GPx immunoblots, a monoclonal antibody, anti-human GPx1 (dilution 1:100 and 1:500 for membrane and cytosol, respectively), produced in mice (sc-130160; Santa Cruz Biotechnology, Inc., Dallas, TX, USA), was used as the primary antibody. In CAT immunoblots, the primary antibody was a monoclonal antibody, anti-human catalase (dilution 1:100 and 1:5000 for membrane and cytosol, respectively), produced in mice (C0979; Sigma-Aldrich, St. Louis, MO, USA). An anti-mouse immunoglobulin G antibody conjugated to horseradish peroxidase, produced in rabbits (A9044, Sigma-Aldrich, St. Louis, MO, USA), was used as the secondary antibody (GPx, dilution 1:1000 and 1:5000 for membrane and cytosol, respectively; CAT, dilution 1:2000 and 1:5000 for membrane and cytosol, respectively). 

To quantify Prx2, RBC membrane (30 μg protein/lane) and cytosol (40 μg protein/lane) samples were submitted to SDS-PAGE under non-reducing conditions. A primary monoclonal antibody, anti-human Prx2 (dilution 1:5000 in 5% low-fat milk and 0.1% Tween 20 in TBS, pH 7.4), produced in mice (MA0144; AbFrontier, Seoul, South Korea), was used. The secondary antibody was the same as that used for CAT and GPx immunoblots (dilution 1:5000 in 5% low-fat milk and 0.1% Tween 20 in TBS, pH 7.4, for both cytosol and membrane samples).

The signals and images from the immunoblots were detected and acquired by the ChemiDoc Touch Imaging System (BIO-RAD, Hercules, CA, USA) using an enhanced chemiluminescence (ECL) substrate (WesternBright ECL horseradish peroxidase Substrate; Advansta, San Jose, CA, USA). The amounts of CAT, GPx, and Prx2 were determined using image analysis software (Image Lab version 5.3.1; BIO-RAD, Hercules, CA, USA).

### 2.6. Total Glutathione and Oxidized Glutathione

Total GSH and GSSG levels were measured in cytosol samples using a spectrophotometric method according to Griffith [38]. Glutathione reductase, in the presence of NADPH, reduces the chromogen DTNB to TNB, which was measured at 412 nm, allowing for the evaluation of total GSH. For GSSG quantification, the same method was applied, but a pre-incubation (1 h, 4 °C) with 2-vinylpiridine (a reduced GSH masking reagent) was performed. The reduced GSH levels were calculated by the difference between total GSH and GSSG levels. The GSH/GSSG ratio was calculated as an indicator of the redox balance within the cell.

### 2.7. Total Antioxidant Status and Ascorbic Acid

TAS and AA concentrations were measured simultaneously, in plasma and cytosol samples, using an adapted spectrophotometric assay [39]. Briefly, a solution of acetate buffer (300 mM, pH 3.6), ferric iron (20.0 mM), and tripyridyltriazine (TPTZ, 10.0 mM) (10:1:1 mL, respectively) was added to each sample; a colored Fe^2+^-TPTZ complex was formed. This complex was also formed by reaction with AA, indirectly allowing for the evaluation of AA concentrations by comparing the reaction mixtures with and without ascorbate oxidase (an enzyme that selectively degrades AA); the difference between them was due, specifically, to AA. TAS and AA concentrations were obtained by using standard samples of ferrous ions in known concentrations (absorbance change at 593 nm; stoichiometric factor of 2.0 for AA). 

### 2.8. Lipid Peroxidation

LPO was determined in plasma and membrane samples using the thiobarbituric acid test, as described by Mihara et al. [40] with modifications, as previously reported [10].

### 2.9. Membrane-Bound Hemoglobin

The MBH was measured in membrane samples after protein dissociation with Triton X-100 (5% *w*/*v* in Dodge buffer) using a spectrophotometric method, as described previously [10]. 

### 2.10. Statistical Analysis

IBM SPSS Statistics 29 for Windows (SPSS Inc., Chicago, IL, USA) was used for the statistical analysis. The normality distribution of continuous data was determined by the Shapiro–Wilk test. Considering that most of the studied parameters presented non-Gaussian distribution, the data are presented as median values (inter-quartile range). For frequency analysis between groups in the categorical variables, such as sex, the Pearson Chi-Square test was used. For continuous variables, the differences between groups were evaluated using the non-parametric Kruskal–Wallis H test, and, when statistical significance was achieved, the Mann–Whitney U test was used to make single comparisons (two groups). The relationships between sets of data were assessed using Spearman’s rank correlation coefficient. To determine the independent predictors of the antioxidant/OS biomarkers in plasma, a multivariate linear regression analysis was performed using stepwise selection, and independent variables were excluded from the predictive models when the collinearity index was over 15; nonparametrically distributed data were previously transformed to follow normality in distribution according to Templeton, G.F.A. [41]. Statistically significant results were achieved when *p* < 0.05.

## 3. Results

Unspl HS and SCD patients, compared to controls, presented lower levels of RBC, Hb, and HCT, and higher RDW, reticulocyte (percentage and count), RPI, and total bilirubin levels, as shown in Table S1 of Supplementary Materials. Unspl HS also presented increases in MCHC and cryohemolysis percentage compared to the control group.

Spl HS patients showed similar levels to controls in the studied parameters, except for higher levels of MCHC, RDW, and cryohemolysis percentage (Table S1). 

In β-thal patients, we observed decreases in Hb, HCT, MCV, MCH, MCHC, and RPI, and higher RBC, RDW, reticulocyte count, total bilirubin, and cryohemolysis percentage compared to the control group (Table S1).

Concerning the OS biomarkers and the antioxidant defenses in plasma, we found that, compared to controls, LPO was higher in all anemias (Table 1). AA was increased in SCD, as well as GPx activity, while CAT activity was higher in spl HS and SCD patients.

**Figure 1:**
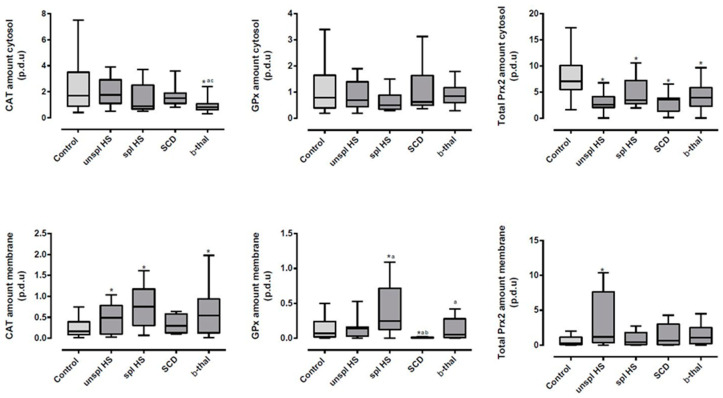
Catalase, glutathione peroxidase, and peroxiredoxin 2 amounts in the red blood cell cytosol and membrane of control, hereditary spherocytosis (unsplenectomized and splenectomized), sickle cell disease, and β-thalassemia groups. Data are presented as median (interquartile range). * *p* < 0.05 vs. control; ^a^ *p* < 0.05 vs. unsplenectomized HS; ^b^ *p* < 0.05 vs splenectomized HS; ^c^ *p* < 0.05 vs. sickle cell disease. β-thal, β-thalassemia; CAT, catalase; GPx, glutathione peroxidase; HS, hereditary spherocytosis; p.d.u., procedure defined unit; Prx2, peroxiredoxin 2; SCD, sickle cell disease; spl, splenectomized; unspl, unsplenectomized.

In RBC cytosol, compared to controls, TAS and AA were increased for all NIHAs; CAT activity was increased in all anemias, except for spl HS, while GPx activity was only higher in SCD (Table 1). Total cytosol Prx2 amount was reduced in all NIHAs (Figure 1). For β-thal patients, compared to the control group, the cytosol CAT amount was lower (Figure 1) and cytosol GPx activity was decreased, together with a higher amount of reduced GSH (Table 1). 

In the RBC membrane, LPO and MBH were increased in all NIHAs compared to controls (Table 1). No differences were found for CAT activity (Table 1), but CAT amount was increased in all NIHAs, except in SCD, compared to controls (Figure 1). The GPx amount was higher in spl HS patients and almost no GPx was observed bound to the membrane in SCD (Figure 1). Only unspl HS patients presented a higher amount of total Prx2 linked to the membrane compared to the control group (Figure 1). 

**Figure 2:**
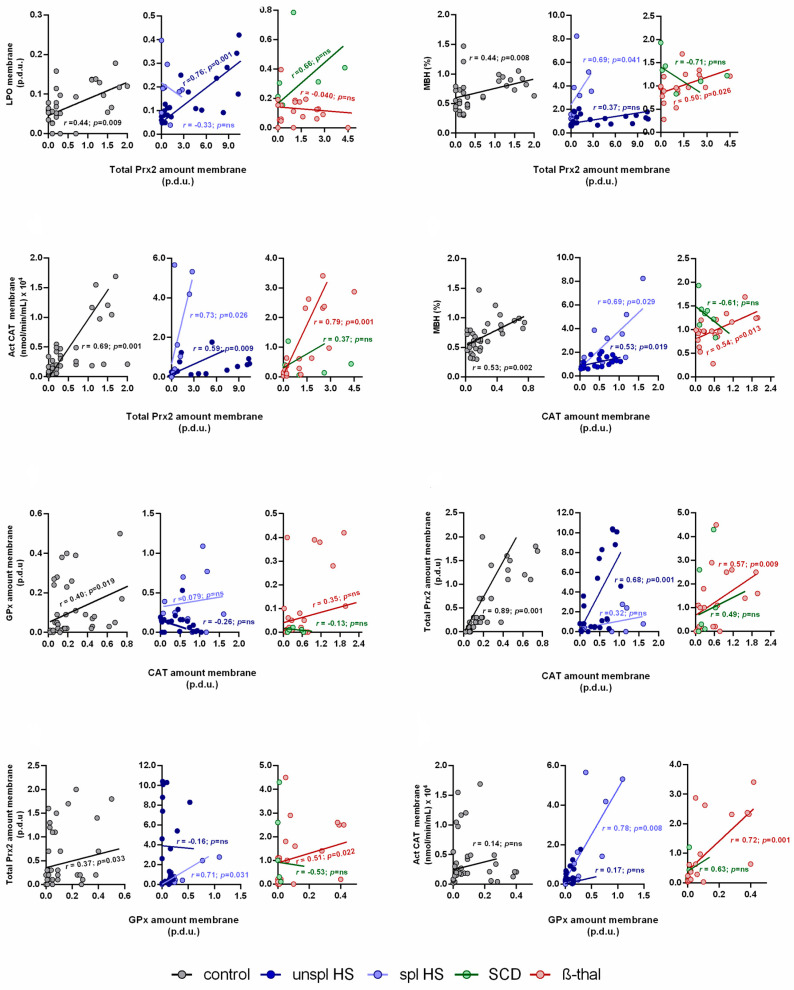
Correlations between oxidative stress markers and antioxidant enzymes in the red blood cell membrane for control, hereditary spherocytosis (unsplenectomized and splenectomized), sickle cell disease, and β-thalassemia groups. Spearman’s rank correlation coefficient was used to evaluate the relationships between sets of data; *p* < 0.05 was considered statistically significant. β-thal, β-thalassemia; Act, activity; CAT, catalase; GPx, glutathione peroxidase; HS, hereditary spherocytosis; LPO, lipid peroxidation; MBH, membrane-bound hemoglobin; ns, non-significant; p.d.u., procedure defined unit; Prx2, peroxiredoxin 2; SCD, sickle cell disease; spl, splenectomized; unspl, unsplenectomized.

The relationships between the antioxidant defenses and the OS biomarkers in the plasma, cytosol, and membrane were analyzed. In controls, we observed several positive correlations between the OS biomarkers and antioxidant defenses (Figure 2 and Table 2): MBH correlated positively with LPO (*r* = 0.50) and both were positively correlated to the binding of both CAT and Prx2 to the membrane (*r* = 0.53, *r* = 0.44 and *r* = 0.53, *r* = 0.44, respectively); cytosol TAS was positively correlated with membrane LPO, MBH, CAT, and Prx2 amounts (*r* = 0.41, *r* = 0.50, *r* = 0.44, and *r* = 0.35, respectively). 

Negative correlations were found between plasma LPO and cytosol AA (*r* = −0.40), and cytosol TAS was inversely correlated with plasma CAT activity (*r* = −0.40), but both plasma LPO and CAT activity were positively correlated (*r* = 0.34) (Table 2). Also, in the cytosol, AA was inversely correlated with CAT activity (*r* = −0.35) but positively correlated with TAS (*r* = 0.56) (Table 2). In the membrane, a positive correlation between Prx2 and CAT amounts (*r* = 0.89) (and CAT activity, *r* = 0.69) was also found, as well as positive correlations between GPx and CAT (*r* = 0.40) and GPx and Prx2 amounts (*r* = 0.37) (Figure 2).

In unspl HS, we observed, in the membrane, positive correlations between LPO and both CAT (*r* = 0.44) and Prx2 amounts (*r* = 0.76) and between MBH and CAT amount (*r* = 0.53) (Figure 2 and Table 2). CAT membrane linkage was positively associated with Prx2 binding (*r* = 0.68) in these patients, and a negative correlation between Prx2 amounts in the cytosol and in the membrane was also found (*r* = −0.52) (Figure 2 and Table 2). 

Spl HS patients presented positive correlations between GPx amount and CAT activity in the membrane (*r* = 0.78) and between CAT amount in the membrane and GPx activity in the cytosol (*r* = 0.81) (Figure 2 and Table 2). Plasma LPO was positively correlated with CAT activity in plasma in these patients (*r* = 0.79) (Table 2).

In SCD, we found positive correlations between LPO and both TAS (*r* = 0.86) and AA in the cytosol (*r* = 0.79); also, MBH was inversely correlated to LPO (*r* = −0.93) and to cytosol TAS (*r* = −0.86) (Table 2). 

Significant positive associations, in the membrane, between MBH and Prx2 (*r* = 0.50), MBH and CAT amount (*r* = 0.54), and Prx2 and CAT amount/activity (*r* = 0.57 and *r* = 0.78, respectively) were observed in β-thal patients (Figure 2). GPx was also detected in the β-thal erythrocyte membrane in association with the increases in Prx2 (*r* = 0.51) and CAT activity (*r* = 0.72) (Figure 2). 

Finally, linear multiple regression analysis was employed to assess which of the studied parameters in the plasma, cytosol, and membrane were individual predictors of the levels of plasma biomarkers; the statistically significant multivariate regression models are depicted in Table S2.

In controls, we found regression models for the prediction of plasma LPO and CAT activity. In unspl and spl HS patients, regression models for the prediction of plasma TAS and LPO and for the prediction of plasma TAS and CAT activity were observed, respectively. Plasma TAS, LPO, and CAT activities were predicted by regression models in both SCD and β-thal groups (Table S2).

## 4. Discussion

By focusing on the erythrocyte antioxidant system and specific OS biomarkers in healthy individuals and in distinct NIHA patients, we aimed to clarify the interplay between some key players of the RBC antioxidant system, studying the erythrocyte cytosol and membrane fractions separately.

### 4.1. Healthy Individuals 

Besides Hb, we found that CAT, GPx, and Prx2 bound to the RBC membrane (Figure 1 and Table 2). CAT was detected in all individuals, but GPx1 and Prx2 were only observed in 30 (88.2%) and 28 (82.4%) subjects, respectively; 27 (79.4%) presented all three enzymes in the membrane. Some authors [5,13,42,43,44,45] also reported the binding of one or more of these enzymes to the RBC membrane. These enzymes, although localized in the cytosol, can bind to the membrane; this linkage may be part of a mechanism by which the RBC protects the membrane from oxidative injuries in response to changes within the RBC itself and/or its environment. 

In fact, the binding of cytosolic proteins to the RBC membrane, namely Hb, has been recognized as a mechanism of cell regulation and signaling in cells deprived of nuclei and organelles [1,44,46,47]. For Hb, specifically, it was demonstrated that this protein is capable of reversible membrane linkage when in low-oxidation states, but as OS increases, heme becomes increasingly oxidized, leading to the irreversible binding of Hb to the membrane, in the form of hemichromes [1], thus triggering band 3 clustering and signalizing the cell for eryptosis [48,49,50,51]. 

MBH correlated positively with LPO (*r* = 0.50) (Table 2), in agreement with the literature [52,53,54] stating that the increase in OS induces the irreversible linkage of Hb to the membrane, which in turn favors membrane LPO. Therefore, both LPO and MBH appear to be membrane OS biomarkers; moreover, these biomarkers were correlated with the (increased) amount of CAT (*r* = 0.53, for both) and of Prx2 in the membrane (*r* = 0.44 for both) (Table 2), suggesting that the linkage of these enzymes might be a physiological response to counteract oxidative injuries. In fact, other studies have proposed that Prx2 and CAT binding might be involved in the protection of the RBC membrane against LPO [10,55,56]. 

As reported elsewhere [43,51], the membrane linkage of both Hb [51] and Prx2 [43], and possibly CAT [45], occurs through the N-terminal cytoplasmic domain of band 3. Indeed, it has been proposed [57] that CAT forms a complex with Prx2 in the cytosol in response to OS; thus, CAT may not attach directly to band 3 but instead through Prx2. Nonetheless, our results are in agreement with these previous studies, as suggested by the positive correlation between Prx2 and CAT amounts (*r* = 0.89) (and CAT activity, *r* = 0.69) in the membrane (Table 2).

We found positive correlations between MBH and Prx2 (*r* = 0.44) and CAT amounts (*r* = 0.53). However, Bayer et al. [13] reported that Prx2 competes with Hb for the same binding site in the RBC membrane, and our group has also found some evidence of the same [5]. It is possible that this competition may occur and be detected only in environments with high OS; actually, in both referred studies, the RBCs were subjected to increased OS conditions. Therefore, in the RBCs of healthy individuals (our control group), it is reasonable to hypothesize that the competition for the binding site to band 3 is not observed when the amounts of both Hb and Prx2 that bind to the membrane are (relatively) low and thus not enough to show that competition. 

As for GPx, the only enzyme for which the binding site to the RBC membrane is still unknown, it may have a distinct membrane linkage site. We found that GPx’s membrane linkage was not correlated with MBH or LPO level (Figure 2), contrary to the translocation of Prx2/CAT to the membrane that appears to be driven by OS injury level, as suggested by the correlations with MBH and LPO (Table 2). However, we found positive correlations for the GPx amount with both CAT (*r* = 0.40) and Prx2 amounts (*r* = 0.37) in the membrane (Table 2); therefore, it is also possible that GPx forms a complex with Prx2 and/or CAT, and, whenever needed for protection of the RBC membrane, is recruited with either of them. We hypothesize that steady-state healthy RBCs face low levels of OS exposure, far from the OS conditions used in in vitro studies or in pathologic conditions. Nonetheless, our data support that, as OS increases, the oxidative membrane injuries increase (LPO/MBH), followed by the activation of the antioxidant defenses in the cytosol and the recruitment of Prx2, CAT, and GPx to the membrane, as suggested by the observed strong positive correlations between TAS in the cytosol and membrane LPO, MBH, CAT, and Prx2 amounts (*r* = 0.41, *r* = 0.50; *r* = 0.44, and *r* = 0.35, respectively) (Table 2). 

Moreover, in the cytosol, AA was inversely correlated with CAT activity (*r* = −0.35) but positively correlated with TAS (*r* = 0.56) (Table 2), which might indicate that AA takes a more prominent role as an antioxidant defense, in an effort to compensate the “loss” of cytosol CAT to the membrane in response to OS increase (Figure 1). This shows that, when facing oxidative injuries at the membrane, erythrocytes trigger the antioxidant defense mechanisms in the cytosol and membrane to counteract oxidative injuries that may ultimately result in cell removal.

Furthermore, we found that plasma LPO was negatively correlated with cytosol AA (*r* = −0.40) and cytosol TAS was inversely correlated with plasma CAT activity (*r* = −0.40), but both plasma LPO and CAT activity were directly correlated (*r* = 0.34). These findings suggested that, in healthy individuals, the lowest circulating levels of LPO were observed for subjects with the highest RBC cytosol antioxidant activity, which is in accordance with the concept that “healthy” RBCs act as a major regulator of the organism’s redox status through its powerful antioxidant system and the inherent fact of reaching all tissues and cells [58,59,60]. In agreement with this hypothesis were the regression models (Table S2) showing that plasma LPO was dependent on cytosol TAS and CAT amounts (R^2^ = 0.68, *p* < 0.001) and that plasma CAT activity was independently predicted by cytosol TAS and GPx amounts (R^2^ = 0.58, *p* < 0.001). Indeed, these are just some examples of the influence of antioxidant/OS biomarkers present in the plasma, cytosol, and membrane on circulating parameters, since not only in healthy individuals but also in the pathological conditions, we observed signs of feedback between RBCs and plasma (Table S2).

### 4.2. Hereditary Spherocytosis (Unsplenectomized Patients)

In unspl HS patients, we found common features of this NIHA, namely, anemia, jaundice, increased RPI (Table S1) [61,62], and increased erythrocyte fragility (cryo-hemolysis test), due to the altered RBC membrane structure [16,19].

The HS RBCs were clearly responding to OS (increased plasma LPO, Table 1), as shown by the high values of cytosol TAS, AA, and CAT activity (Table 1), presenting oxidative changes mainly in the membrane. 

The MBH and membrane LPO levels were significantly increased compared to controls; however, compared to the other NIHAs, they presented some of the lowest values (specially LPO), which could be associated with the significant increases in CAT and Prx2 linked to the membrane (Table 1 or Figure 1). Our data are in accordance with other studies [10,55,56] suggesting that CAT and Prx2 might confer protection to the RBC membrane in these patients. In addition, this hypothesis is further supported by the positive correlations we found between membrane LPO with both CAT (*r* = 0.44) and Prx2 amounts (*r* = 0.76), and between MBH and CAT amount (*r* = 0.53) (Table 2 and Figure 2).

The high levels of OS markers in the membrane could also result, at least in part, from the high rate of metHb formation, which is favored by the increased intracellular Hb concentration levels (high MCHC, Table S1), as described elsewhere [17,18]. According to Welbourn E.M. et al. [1], the binding of metHb to the membrane can also be a consequence of OS that induces protein degradation, which could also explain the high levels of OS markers in these patients. 

We also found the highest levels of Prx2 bound to the membrane, and it was the only group of patients presenting a negative correlation between Prx2 amounts in the cytosol and membrane (*r* = −0.52), suggesting translocation of Prx2 from the cytosol to the membrane (Figure 1 and Table 2). CAT membrane linkage, as occurred in the controls, also appeared to be associated with Prx2 binding (positive correlation, *r* = 0.68, Figure 2). Prx2 and CAT seem to be the key enzymes involved in the mechanism of protection of RBCs from unspl HS patients, as no significant alterations were observed for GPx. We hypothesize that this may be related to the fact that the binding site of Prx2 and CAT is band 3 [43,57], and the pathophysiology of HS is specifically an alteration in the membrane protein complex containing this transmembrane protein [15].

### 4.3. Hereditary Spherocytosis (Splenectomized Patients)

The patients with HS who underwent splenectomy presented changes that were in agreement with this standard treatment [19], with the correction of anemia and normalization of bilirubin and reticulocyte values (Table S1). However, as the protein defect in the RBC membrane remained, spherocytes still persisted in circulation but they had longer half-lives, explaining the highest value of cryo-hemolysis observed, as compared to controls (Table S1). Since these cells survived longer, the oxidative changes were irreversible and cumulative; thus, RBCs from spl patients present increased fragility due to the altered membrane structure [10,63,64]. 

As observed for the unspl patients, the spl patients did not show the correlations that were found in the cytosol for the control group (Table 2), also suggesting that, in spl HS RBCs, the main oxidative changes are observed at the membrane.

A significant increase in GPx amount in the membrane, only observed in spl patients, together with an increase in CAT amount, are probably the response to the very high LPO levels (Figure 1 and Table 1). Actually, both enzymes are reported to prevent LPO in the RBC membrane [9,10], and GPx is described as particularly important in hydroperoxide scavenging [4,9]. Furthermore, GPx seems to be paramount and acts in cooperation with CAT, as suggested by the observed positive correlations between GPx amount in the membrane and CAT activity (*r* = 0.78) (Figure 2) and, also between CAT amount in the membrane and GPx activity in the cytosol (*r* = 0.81) (Table 2).

Contrary to what we observed in unspl patients, there was no increase in Prx2 binding to the membrane in spl HS accompanying its decrease in the cytosol (Figure 1). This could be explained by the significantly high MBH found in spl patients and by the fact that Prx2 and Hb compete for the same binding site in the membrane [13]. In all other anemias, we also observed an increase in MBH, but those values were lower than in spl HS (Table 1); thus, Prx2 is still able to bind to the membrane, while in spl patients, there are just very few binding sites available for Prx2. Compared to the other anemias, RBCs from spl HS patients had longer half-lives and thus more oxidative injuries were accumulated [1], which, we believe, was reflected in the higher amount of MBH that we observed. This, together with the reported significant increase in GPx, may be indicative that, in fact, GPx and Prx2 do not share the same membrane-binding location, as proposed above. 

Most likely, due to the accumulation of oxidative damage in the erythrocyte membrane of spl patients, the plasma antioxidant defenses take a more prominent role in counteracting the significantly high LPO, as seen by the increase in CAT activity in plasma and by the positive correlation between these two parameters (*r* = 0.79) (Table 1 and Table 2). Interestingly, only for the spl HS group, no regression model was found to predict plasma LPO (Table S2).

### 4.4. Sickle Cell Disease

Patients with SCD presented the most severe anemia of all the studied NIHAs, as shown by the lowest RBC, Hb, and HCT levels (Table S1) [25,26]. Significantly high RPI and reticulocyte levels were found, compared to the control group, but not as high as in unspl HS, despite the higher severity of the anemia, showing that, in SCD, the erythropoietic response to anemia is impaired and not as effective as in HS patients (Table S1). Cryo-hemolysis was similar to controls, showing that sickle RBCs were more resistant to lysis than in other NIHAs, as reported by Tatum V. and Chow C. [65]. 

SCD patients showed clear signs of increased OS, even more enhanced than in unspl HS patients, as shown by the very high levels of MBH and LPO (membrane and plasma) and of TAS, AA, CAT, and GPx activities (cytosol and plasma) compared to the control group (Table 1). Notably, SCD was the only of the studied anemias in which GPx activity was significantly increased in both plasma and cytosol (Table 1), in accordance with other reports [24,26]. However, the debate carries on, since opposite data have also been reported [66,67], and one study associated the decrease in GPx activity with low levels of Se [68]. We believe that this controversy might result, at least in part, from the difficulty in studying representative populations, given the prevalence of the disease. 

The sickle RBCs, as compared to the other NIHAs, presented lower amounts of the antioxidant enzymes linked to the membrane. CAT and Pxr2 showed similar levels, compared to controls, while in the other anemias, the membrane amounts of these enzymes were, in most cases, significantly increased; furthermore, GPx membrane binding was almost nonexistent (Figure 1). We hypothesize that the lower linkage of CAT, GPx, and Prx2 to the RBC membrane, in these patients, might be due to conformational modifications induced by HbS. In fact, it was described [27,69,70] that HbS, due to its higher propensity for oxidation and irreversible linkage to the membrane compared to HbA, causes changes in membrane structure and in cytoskeleton proteins, such as in band 3 [71,72,73] and spectrin [74]. Furthermore, some lipids and membrane proteins responsible for maintaining lipid organization may be damaged in SCD [75]. Thus, the normal lipid organization and composition of the plasma membrane are lost, leading to the reorganization of membrane lipids and the externalization of phosphatidylserine, which is involved in the vaso-occlusion process characteristic of SCD [67,69,70,73]. Since band 3 is the membrane-binding site of Prx2 (and CAT), as referred previously, this might explain the apparent decrease in membrane linkage of these enzymes. 

Regarding GPx, the exact binding site to the membrane is not known, but we believe that it is also affected by structural changes caused by HbS due to the observed impediment of GPx linkage (Figure 1). Our findings may also be empirical evidence that the localization site of GPx to the membrane is not band 3, since Prx2 and CAT bound less but were not completely absent from the membrane. On the other hand, as proposed recently, for SCD patients [24,25], the faster auto-oxidation of HbS compared to normal Hb will result in higher generation of ROS, shown by the highest LPO (and the inverse correlation between MBH and LPO, *r* = −0.93). Thus, high OS levels may lead to the consumption or inactivation of the antioxidant enzymes, explaining their low levels in the membrane. 

Our data suggest that, in spite of the cell’s efforts to counteract oxidative damage, as shown by the correlations between LPO and cytosol TAS/AA (*r* = 0.86 and *r* = 0.79), the highest membrane LPO value probably results from the reduced amount of antioxidant enzymes present in the membrane, especially of GPx, which has a main role in RBC membrane protection against LPO [4,10,42,76]. Some authors [25,26,70,72,75] believe that membrane OS-induced changes contribute to the pathophysiology of SCD; this evident lack of antioxidant enzyme binding to the RBC membrane may, actually, have a major contribution to the higher clinical severity observed in SCD patients, as compared to the other studied NIHAs (Table S1). 

### 4.5. β-Thalassemia

β-thal patients showed the common features of this type of anemia [77], namely, significant decreases in Hb concentration, MCV, and MCHC, and a normal number of RBCs, which was the highest in all of the studied groups (Table S1). Bilirubin concentration and reticulocyte count were increased, compared to controls, but were lower than in unspl HS and SCD. In fact, RPI was significantly lower than in controls (and the other anemias), denoting the ineffective erythropoiesis due to apoptosis of erythroid precursors, as described elsewhere [78,79]. 

β-thal erythrocytes showed clear changes in OS biomarkers. Accordingly, in the cytosol, there were increases in TAS and AA, as well as reductions in GSH, GPx, and CAT activities and in CAT and Prx2 amounts (Figure 1 and Table 1). In the membrane, we observed higher levels of LPO and MBH (Table 1). The review by Awadallah S. [80] describes controversial reports about OS biomarkers as being either increased or decreased in thalassemia, except for the consensual increase in LPO, also found in the present work. We must note that we studied CAT and GPx activities (and amounts) in the RBC cytosol and membrane fractions separately in a β-thal minor cohort, which was not performed in the reported studies; therefore, a direct comparison is not possible.

The most distinctive changes in β-thal, which were not found in the other NIHAs, were the decrease in cytosol GPx activity, increase in GSH, but no significant change in GSH/GSSG ratio compared to the control group (Table 1). Considering that the GPx amount in the cytosol was similar to controls (Figure 1), it appears that its activity is impaired, resulting in the accumulation of GSH. As thoroughly discussed elsewhere [81], there are several works hypothesizing about the decrease in GPx activity in β-thal, including one [82] that associated decreased levels of Se with lower GPx activity. Since we found significantly higher GPx activity in the cytosol and plasma (Table 1) of SCD patients and it has been extensively reported [68,83,84,85] that these patients have Se deficiency, we believe that low Se levels may not be the major explanation in the case of β-thal patients. We did not evaluate Se levels in our population of β-thal minor patients, thus, undoubtedly, further studies are needed to address this question.

Another unique finding in β-thal patients was the clear translocation of CAT, as suggested by the significant decrease in CAT amount in the cytosol and its significant increase in the membrane (Figure 1). 

As found in RBCs from healthy individuals, we observed positive correlations between MBH and Prx2 (*r* = 0.50) and CAT amounts (*r* = 0.54) in the membrane, and between Prx2 amount in the membrane and CAT membrane amount and activity (*r* = 0.57 and *r* = 0.78, respectively) (Figure 2), further supporting that the linkage of Hb, Prx2, and CAT to the membrane is intertwined. Similarly to controls, GPx was detected in the β-thal erythrocyte membrane, showing positive correlations with Prx2 amount (*r* = 0.51) and CAT activity in the membrane (*r* = 0.72) (Figure 2).

Interestingly, although LPO was increased, compared to controls (Table 1), it was not correlated with Hb, CAT, and Prx2 linkage to the membrane (Figure 2 and Table 2). Thus, in β-thal RBCs, these enzymes may not be the main players in counteracting LPO effects, as proposed for healthy RBCs, or these results may reflect a dysfunction in the antioxidant response, specific to β-thal. 

### 4.6. General Conclusions 

In most of the studied anemias, we observed an increase in plasma OS biomarkers, such as LPO and antioxidant enzyme activities (Table 1), showing that the metabolic activity of these pathological RBCs was compromised and they were not able to act as efficient scavengers of systemic ROS. This was especially evident for SCD and spl HS, which presented with much more OS-induced membrane injuries (Table 1). The increases in membrane LPO and MBH, observed in all NIHAs, were usually associated, and they appear to explain the rises in cytosol TAS, AA, and CAT activities (Table 1 and Table 2, and Figure 2), probably as an effort for counteract oxidative injuries. In a previous work [86], we reported that AA has an important contribution to RBC cytosol antioxidant defenses. 

As referred above, the antioxidant response of cytosol GPx (and GSH) is controversial and appears to be dependent on the type of anemia (Figure 1 and Table 1) and on the nature of the cohorts studied herein. It is important to note that, given the prevalence of these anemias, in some cases, it is difficult to obtain a representative case number population for study, as occurred for SCD.

Another common feature in the studied anemias was that the Prx2 amount in the cytosol was significantly lower compared to controls. In the case of unspl HS, it can be explained by the translocation of Prx2 to the membrane (Figure 1). However, in SCD, β-thal, and spl HS, the decrease in PRx2 may only be in part explained by its delocalization to the membrane, as only trends for higher amounts of membrane Prx2 were found (Figure 1). There is clearly a distinct response regarding Prx2 according to each underlying anemia. Due to different genetic mutations, changes in the erythroid cell antioxidant capacity may start early on, along with erythropoiesis [87]. Very recently, by studying circulating reticulocytes from patients with these types of NHIA, we found that only in the reticulocytes from unspl HS were the mRNA levels of Prx2 positively correlated with reticulocyte maturity indices [88]. In fact, the importance of Prx2 in the development of erythroid precursor cells has been reported in other studies [4,89,90]. 

In RBCs from healthy individuals, and in NIHA RBCs, the linkage of Prx2, CAT, and GPx to the membrane is probably the primary protective mechanism against oxidative membrane injury. However, due to the distinct pathophysiologies of the anemias, the protective effect of these enzymes is affected differently. In both β-thal and unspl HS, our data suggested translocation from the cytosol to the membrane of the antioxidant enzymes CAT and Prx2 (Figure 1), which indicates that, in these anemias, RBCs are recruiting antioxidant defenses to the membrane. In RBCs from SCD patients, the changes in membrane structure, cytoskeleton, and lipid organization due to HbS sickling may alter the interactions between the RBC membrane and antioxidant enzymes, explaining the different membrane antioxidant profile, with a reduced amount of each enzyme bound to the membrane (Figure 1). Interestingly, we showed that SCD individuals presented an immature reticulocyte population with high mRNA levels of *CAT*, *GPX1*, and *PRDX2* [88]. These data suggest that, although reticulocytes from SCD patients present with the potential for increased amounts of CAT, GPx, and Prx2 in mature erythrocytes, this is not reflected in the binding of these enzymes to the membrane.

The spl HS patients, with RBCs presenting almost normal longevity, showed an unmatched antioxidant profile of which the most striking change was the accumulation of MBH, CAT, and GPx in the membrane (Table 1). 

In the present work, we undoubtedly show that all of the studied NIHAs are associated with increased OS. However, the OS-induced changes and the antioxidant system responses are quite different, which is most likely related to the different anemia etiologies. These different RBC antioxidant defense system responses can contribute to, or even trigger, the very different clinical patterns/symptoms seen in distinct NIHA patients.

## Figures and Tables

**Table 1:** Oxidative stress markers and enzyme activities in the plasma, cytosol, and membrane of control, hereditary spherocytosis (unsplenectomized and splenectomized), sickle cell disease, and β-thalassemia groups.

	Control (*n* = 34)	unspl HS (*n* = 22)	spl HS (*n* = 10)	SCD (*n* = 7)	β-Thal (*n* = 20)
PLASMA					
TAS (mM)	0.35 (0.27–0.48)	0.35 (0.28–0.40)	0.49 (0.35–0.57) ^a^	2.89 (2.21–3.09) *	0.34 (0.30–0.47)
AA (mM)	0.005 (0.002–0.010)	0.007 (0.003–0.018)	0.006 (0.004–0.017)	0.079 (0.024–0.115) *	0.007 (0.002–0.011)
LPO (p.d.u.)	5.76 (4.93–7.81)	9.05 (4.93–11.51) *	9.04 (6.58–12.54) *	1.63 (1.25–2.19) *	7.80 (6.58–10.49) *
CAT activity (nmol/min/mL)	69 (55–108)	82 (60–102)	118 (104–204) *^a^	998 (899–2526) *^ab^	55 (43–108) ^bc^
GPx activity (mU/mL) × 10^3^	4.01 (2.67–6.78)	5.11 (1.93–8.46)	6.20 (1.13–12.97)	4.81 (1.36–7.83)	4.66 (2.60–7.09)^c^
CYTOSOL					
TAS (mM)	1.93 (1.64–2.22)	2.71 (2.30–3.07) *	3.06 (2.46–3.15) *	2.89 (2.21–3.09) *	2.57 (2.32–3.13) *
AA (mM)	0.018 (0.013–0.034)	0.082 (0.039–0.142) *	0.050 (0.028–0.130) *	0.079 (0.024–0.115) *	0.052 (0.023–0.087) *^a^
CAT activity (nmol/min/mL) × 10^4^	1.10 (0.98–1.22)	1.28 (1.03–1.46) *	1.13 (1.08–1.46)	1.63 (1.25–2.19) *	1.39 (1.23–1.87) *
GPx activity (mU/mL)	702 (565–863)	541 (461–818)	686 (362–896)	998 (899–2526) *^ab^	536 (303–652) *^c^
GSSG (µM)	3.72 (2.09–7.47)	3.51 (1.82–6.11)	3.72 (1.82–5.77)	4.81 (1.36–7.83)	5.51 (3.34–8.53)
GSH (µM)	12.5 (8.4–18.4)	14.3 (12.0–18.7)	15.8 (13.4–18.4)	17.4 (12.9–19.1)	20.7 (17.8–29.4) *^ab^
GSH/GSSG	3.8 (1.4–8.3)	5.4 (2.0–7.8)	5.8 (2.5–9.1)	3.1 (1.8–12.8)	4.1 (2.3–8.0)
MEMBRANE					
LPO (p.d.u.) × 10^−2^	6.2 (3.3–11.5)	10.6 (7.9–19.3) *	19.1 (9.9–22.5) *	30.5 (21.2–40.9) *^b^	11.8 (5.5–17.6) *^bc^
MBH (%)	0.66 (0.49–0.83)	1.16 (0.76–1.52) *	3.38 (1.19–4.19) *^a^	1.34 (1.10–1.43) *	0.97 (0.86–1.21) *^abc^
CAT activity (nmol/min/mL) × 10^3^	2.1 (1.5–4.9)	2.9 (1.7–6.8)	12.3 (1.7–44.7)	2.7 (0.6–6.3)	4.9 (0.9–23.2)

Data are presented as median (interquartile range). * *p* < 0.05 vs. control; ^a^ *p* < 0.05 vs. unsplenectomized HS; ^b^ *p* < 0.05 vs. splenectomized HS; ^c^ *p* < 0.05 vs. sickle cell disease. AA, ascorbic acid; β-thal, β-thalassemia; CAT, catalase; GPx, glutathione peroxidase; GSH, reduced glutathione; GSSG, oxidized glutathione; HS, hereditary spherocytosis; LPO, lipid peroxidation; MBH, membrane-bound hemoglobin; p.d.u., procedure defined unit; SCD, sickle cell disease; spl, splenectomized; TAS, total antioxidant status; unspl, unsplenectomized.

**Table 2:** Correlations between antioxidant defenses and oxidative stress biomarkers in the plasma, cytosol, and membrane for control, hereditary spherocytosis (unsplenectomized and splenectomized), sickle cell disease, and β-thalassemia groups.

		Control (*n* = 34)	unspl HS (*n* = 22)	spl HS (*n* = 10)	SCD (*n* = 7)	β-Thal (*n* = 20)
AA cyt vs. Act CAT cyt	*r*	−0.35	0.30	−0.36	0.11	−0.35
	*p*	0.045	ns	ns	ns	ns
TAS cyt vs. AA cyt	*r*	0.56	0.39	0.33	0.68	0.65
	*p*	0.001	ns	ns	ns	0.003
TAS cyt vs. LPO m	*r*	0.41	0.14	0.38	0.86	0.30
	*p*	0.016	ns	ns	0.014	ns
TAS cyt vs. MBH m	*r*	0.50	0.13	0.084	−0.86	0.24
	*p*	0.003	ns	ns	0.014	ns
TAS cyt vs. Act CAT m	*r*	0.54	0.10	−0.44	0.086	0.14
	*p*	0.001	ns	ns	ns	ns
TAS cyt vs. CAT m	*r*	0.44	0.43	0.084	0.75	0.51
	*p*	0.008	ns	ns	ns	0.024
TAS cyt vs. Prx2 m	*r*	0.35	0.30	−0.54	0.60	0.12
	*p*	0.042	ns	ns	ns	ns
LPO m vs. MBH	*r*	0.50	0.25	0.10	−0.93	−0.092
	*p*	0.003	ns	ns	0.003	ns
LPO m vs. CAT m	*r*	0.53	0.44	−0.055	0.68	−0.077
	*p*	0.001	0.048	ns	ns	ns
LPO m vs. AA cyt	*r*	0.27	0.12	0.15	0.79	0.35
	*p*	ns	ns	ns	0.036	ns
CAT m vs. Act GPx cyt	*r*	0.19	0.51	0.81	0.39	0.70
	*p*	ns	0.019	0.005	ns	0.001
Prx2 m vs. Prx2 cyt	*r*	0.27	−0.52	−0.13	−0.38	0.083
	*p*	ns	0.017	ns	ns	ns
TAS p vs. AA p	*r*	0.41	0.35	0.84	0.29	0.12
	*p*	0.017	ns	0.002	ns	ns
LPO p vs. Act CAT p	*r*	0.34	0.25	0.79	0.41	0.26
	*p*	0.049	ns	0.007	ns	ns
LPO p vs. AA cyt	*r*	−0.40	0.015	0.085	−0.68	−0.029
	*p*	0.021	ns	ns	ns	ns
Act CAT p vs. TAS cyt	*r*	−0.40	0.19	0.48	0.16	0.098
	*p*	0.018	ns	ns	ns	ns

Spearman’s rank correlation coefficient was used to evaluate relationships between sets of data; *p* < 0.05 was considered statistically significant. β-thal, β-thalassemia; AA, ascorbic acid; Act, activity; CAT, catalase; cyt, cytosol; GPx, glutathione peroxidase; HS, hereditary spherocytosis; LPO, lipid peroxidation; m, membrane; MBH, membrane-bound hemoglobin; ns, non-significative; p, plasma; Prx2, peroxiredoxin 2; spl, splenectomized; TAS, total antioxidant status; unspl, unsplenectomized; vs, versus.

## Data Availability

The raw data supporting the conclusions of this article will be made available by the corresponding authors upon request. The data are not publicly available due to privacy and ethical reasons.

## Supplementary Materials

The following supporting information can be downloaded at: https://www.mdpi.com/article/10.3390/antiox13060629/s1, Table S1: Hematological and biochemical data for control, hereditary spherocytosis (unsplenectomized and splenectomized), sickle cell disease, and β-thalassemia groups. Table S2: Multiple linear regression models of prediction of plasma’s biomarkers under study, considering all the other evaluated biomarkers in plasma plus RBC’s cytosol and membrane as independent variables, for control, hereditary spherocytosis (unsplenectomized and splenectomized), sickle cell disease and β-thalassemia groups.
