# Supplementary material for: Catalase, Glutathione Peroxidase, and Peroxiredoxin 2 in Erythrocyte Cytosol and Membrane in Hereditary Spherocytosis, Sickle Cell Disease, and β-Thalassemia
Source: Antioxidants (Basel). 2024 May 22;13(6):629. doi: 10.3390/antiox13060629 (PMC11201268; doi:10.3390/antiox13060629)
Supplement: Supplementary file 1 [file antioxidants-13-00629-s001.zip › antioxidants-3000422-supplementary.pdf]

Table S1. Hematological and biochemical data for control, hereditary spherocytosis (unsplenectomized and splenectomized), sickle cell disease, and β-thalassemia groups.

	**Control** (*n* = 34)	**unspl HS** (*n* = 22)	**spl HS** (*n* = 10)	**SCD** (*n* = 7)	**β-thal** (*n* = 20)
RBCs (× 10^{12}/L)	4.81 [4.56–5.10]	4.00 [3.45–4.45] *	4.63 [3.59–5.36]	2.38 [2.20–3.40] *[ab]	5.65 [5.07–5.92] *[abc]
Hb (g/L)	147 [134–152]	123 [113–131] *	138 [111–168]	87 [76–94] *[ab]	112 [104–126] *[bc]
HCT (%)	42 [40–45]	35 [33–36]*	39 [34–48]	24 [21–29] *[ab]	33 [33–39]*[c]
MCV (fL)	88.2 [86.8–89.9]	86.0 [81.7–91.6]	87.9 [82.0–92.8]	90.9 [80.0–110.6]	62.6 [60.1–69.6] *[abc]
MCH (pg)	30.1 [29.2–30.6]	30.4 [29.1–32.7]	31.4 [28.4–32.9]	32.8 [26.3–40.1]	20.0 [19.2–21.9] *[abc]
MCHC (g/dL)	33.9 [33.6–34.4]	35.3 [34.8–36.2] *	35.2 [34.4–36.4] *	35.4 [33.3–36.0]	32.3 [32.2–32.9] *[abc]
RDW (%)	12.7 [12.3–13.1]	16.8 [14.2–18.6] *	14.8 [13.3–17.2] *	19.0 [18.4–21.0] *[ab]	15.9 [15.3–16.6] *[c]
RET (%)	1.20 [0.97–1.44]	5.50 [3.98–7.29] *	1.56 [1.08–2.07] [a]	5.29 [4.33–6.74] *[b]	1.49 [1.00–1.88] [ac]
RET (× 10^9/L)	55 [46–71]	215 [143–263] *	60 [37–86] [a]	177 [136–197] *[b]	77 [51–107] *[ac]
RPI	1.05 [0.79–1.39]	2.25 [1.93–2.79] *	1.18 [0.64–2.0] [a]	1.73 [1.21–2.10] *	0.70 [0.60–0.90] *[ac]
Total Bilirubin (mg/dL)	0.69 [0.54–0.84]	1.56 [1.17–2.57] *	0.90 [0.50–0.96] [a]	2.19 [1.43–4.53] *[b]	0.90 [0.70–1.28] *[ac]
Cryohemolysis (%)	4.0 [2.5–9.4]	19.2 [1.8–25.1] *	29.4 [6.4–42.1] *	4.8 [3.3–9.1] [b]	20.9 [0.8–33.5] *

Data are presented as median (interquartile range). * $p < 0.05$ vs. control; [a] $p < 0.05$ vs. unsplenectomized HS; [b] $p < 0.05$ vs. splenectomized HS; [c] $p < 0.05$ vs. sickle cell disease. β-thal, β-thalassemia; HS, hereditary spherocytosis; Hb, hemoglobin; HCT, hematocrit; MCH, mean corpuscular hemoglobin; MCHC, mean corpuscular hemoglobin concentration; MCV, mean corpuscular volume; PLT, platelets; RBC, red blood cells; RDW, red cell distribution width; RET, reticulocyte; RPI, reticulocyte production index; spl, splenectomized; unspl, unsplenectomized; WBC, white blood cells.

Table S2 – Multiple linear regression models of prediction of plasma's biomarkers under study, considering all the other evaluated biomarkers in plasma plus RBC's cytosol and membrane as independent variables, for control, hereditary spherocytosis (unsplenectomized and splenectomized), sickle cell disease and β-thalassemia groups.

Dependent variable	Model adjusted R^2; *p*	Independent variables (Predictors)	Unstandardized coefficients		Standardized coefficients	t	*p*
			B	Std. error	Beta		
**Control (*n* = 34)**							
LPO p	0.683 <0.001	(constant)	3.905	0.276		11.235	<0.001
		CAT cyt	-0.275	0.062	-0.591	-4.475	<0.001
		TAS cyt	-0.521	0.138	-0.497	-3.767	0.002
Act CAT p	0.580 <0.001	(constant)	5.484	0.328		16.735	<0.001
		TAS cyt	-0.622	0.161	-0.583	-3.872	0.001
		GPx cyt	-0.205	0.069	-0.449	-2.980	0.008
**unspl HS (*n* = 22)**							
TAS p	0.369 0.006	(constant)	0.203	0.045		4.511	<0.001
		Prx2 cyt	0.041	0.013	0.639	3.221	0.006
LPO p	0.282 0.017	(constant)	2.681	2.162		1.240	0.234
		Act GPx Cyt	0.010	0.004	0.571	2.697	0.017
**spl HS (*n* = 10)**							
TAS p	0.622 0.039	(constant)	0.444	0.041		10.751	<0.001
		Act CAT m	-0.106	0.035	-0.835	-3.037	0.039
Act CAT p	0.957 0.004	(constant)	5.297	0.112		47.146	<0.001
		GPx cyt	1.413	0.141	1.305	10.051	0.002
		MBH	0.536	0.119	0.586	4.513	0.020
**SCD (*n* = 7)**							
TAS p	1.000 0.003	(constant)				-714.42	<0.001
		LPO p	0.064	0.000	0.770	272.75	0.002
		LPO m	-0.312	0.002	-0.444	-170.73	0.004
		Prx2 cyt	0.029	0.001	0.111	38.885	0.016
LPO p	0.979 0.011	(constant)	-49.118	5.190		-9.463	0.011
		Act GPx cyt	8.493	0.721	0.864	11.780	0.007
		CAT cyt	-5.078	0.973	-0.383	-5.222	0.035
Act CAT p	1.000 <0.001	(constant)	5.635	0.000		23835	<0.001
		CAT cyt	-1.928	0.000	-1.255	-6488.0	<0.001
		GPx m	-40.224	0.013	-0.586	-3032.5	<0.001
		CAT m	-0.056	0.000	-0.067	-443.90	0.001
**β-thal (*n* = 20)**							
TAS p	0.672 0.002	(constant)	0.059	0.075		0.784	0.456
		LPO p	0.036	0.008	0.842	4.406	0.002
LPO p	0.672 0.002	(constant)	1.378	1.768		0.780	0.458
		TAS p	19.877	4.512	0.842	4.406	0.002
Act CAT p	0.856 0.005	(constant)	8.341	0.730		11.426	<0.001
		TAS cyt	-1.151	0.233	-0.863	-4.946	0.004
		CAT cyt	-0.874	0.317	-0.412	-2.756	0.040

Multivariate linear regression (Stepwise method) was used to estimate the model´s adjusted R square (R^2); $p < 0.05$ was considered statistically significant. β-thal, β-thalassemia; AA, ascorbic acid; Act, activity; CAT, catalase; cyt, cytosol; GPx, glutathione peroxidase; HS, Hereditary Spherocytosis; LPO, lipid peroxidation; m, membrane; MBH, membrane bound hemoglobin; p, plasma; Prx2, peroxiredoxin 2; spl, splenectomized; TAS, total antioxidant status; unspl, unsplenectomized
